# The Dietary Branched-Chain Amino Acids Transition and Risk of Type 2 Diabetes Among Chinese Adults From 1997 to 2015: Based on Seven Cross-Sectional Studies and a Prospective Cohort Study

**DOI:** 10.3389/fnut.2022.881847

**Published:** 2022-05-23

**Authors:** Lianlong Yu, Pengkun Song, Qianrang Zhu, Yuqian Li, Shanshan Jia, Shixiu Zhang, Zhihong Wang, Jian Zhang

**Affiliations:** ^1^National Institute for Nutrition and Health, Chinese Center for Disease Control and Prevention, Beijing, China; ^2^NHC Key Laboratory of Trace Element Nutrition, National Institute for Nutrition and Health, Chinese Center for Disease Control and Prevention, Beijing, China; ^3^Department of Nutrition and Food Hygiene, School of Public Health, Cheeloo College of Medicine, Shandong University, Jinan, China

**Keywords:** nutritional epidemiology, branched chain amino acids, transition, nutrient effects, type 2 diabetes, risk analysis

## Abstract

**Background:**

The situation is grim for the prevention and control of type 2 diabetes (T2D) and prediabetes in China. Serum and dietary branched-chain amino acids (BCAAs) were risk factors for T2D. However, there is a lack of information on trends in consumption of BCAAs and the risk of T2D associated with BCAAs intake, based on nationally representative data in China. Thus, we aimed to comprehensively describe the dietary BCAAs transition and risk of T2D, at a national level among Chinese adults from 1997 to 2015.

**Methods:**

The data sources were the China Health and Nutrition Survey (CHNS) and China Nutrition and Health Survey (CNHS). Cross-sectional data on intake were obtained from CHNS (1997, *n* = 9,404), CHNS (2000, *n* = 10,291), CHNS (2004, *n* = 9,682), CHNS (2006, *n* = 9,553), CHNS (2009, *n* = 9,811), CHNS (2011, *n* = 12,686) and CNHS (2015, *n* = 71,695). Prospective cohort data were obtained CHNS (1997–2015, *n* = 15,508).

**Results:**

From 1997 to 2015, there was a significant decreasing trend in the BCAAs intake of Chinese adults in all subgroups (*P* < 0.0001) except for Leu in 80 or older, and a decreasing trend in the consumption of BCAAs after 40 years old (*P* < 0.05). The mean intake of BCAAs in the population of cohort study was 11.83 ± 3.77g/day. The 95% CI was above the HR of 1.0, when the consumptions were higher than 14.01, 3.75, 6.07, 4.21 g/day in BCAAs, Ile, Leu and Val, based on RCS curves. According to the Cox proportional hazards models, Compared with individuals with BCAAs consumption of 10.65–12.37 g/day, the multivariable-adjusted HR for diabetes was 2.26 (95% CI 1.45 to 3.51) for individuals with consumption of BCAAs more than 18.52 g/day. A statistically significant positive association between BCAAs intake and risk of T2D was observed in males or participants aged 45 years and older, but not in females or participants younger than 45 years.

**Conclusion:**

Our results reveal a trend toward decreased BCAAs intake in Chinese from 1997 to 2015. After 40 years of age, consumption of BCAAs declined with increasing age. Higher BCAAs intake was associated with higher risk of T2D. This relationship is more stable among men and middle-aged and elderly people.

## Introduction

Branched-chain amino acids (BCAAs), including leucine (Leu), isoleucine (Ile), and valine (Val), are essential amino acids for mammals (1) and are supplied considerably from diet. Previous studies have shown that the main food sources of BCAAs in the US population were meat (37%), milk (12%), and fish (8%), while in the Japanese population the main contributors were cereals, potatoes and starches (23–25%), fish and shellfish (21–23%) and meat (14–15%) (2). BCAAs were critical components of dietary protein. Elevations in branched-chain amino acids (BCAAs) associated with numerous systemic diseases, including cancer, type 2 diabetes (T2D), and heart failure (3). Reports since the 1960's have noted that elevations in circulating BCAAs tightly associate with insulin resistance (4).

The prevalence of diabetes in China has increased dramatically in the past two decades (5, 6). Elevated plasma branched chain amino acids (BCAAs) has been implicated in development of insulin resistance and T2D. However, whether consumption of BCAAs contribute to the disease is controversial. Some studies have shown that high intake of BCAAs is associated with an increased risk of T2D (2, 7, 8) and may have adverse effects on the development of IR (9). On the contrary, a study from a Japanese population reported that high intake of BCAAs may be associated with reduced diabetes risk (10). Research in this area has remained relatively limited. Thus, the association between dietary BCAAs and the risk of T2D in Chinese adults is unclear. Also, the quantity of BCAAs intake causing risk of T2D is not clearly defined. It could have significant clinical and public health implications that finding out exact BCAAs consumption threshold values of developing diabetes.

In the past few decades, dietary structure and food intakes of Chinese have undergone substantial changes (11). However, there is a lack of information on trends in BCAAs consumption and the risk of T2D associated with BCAAs intake, based on nationally representative data. Using data from 1997 to 2015 China Health and Nutrition Survey (CHNS) and China Nutrition and Health Survey (CNHS), the current study was aimed to systematically describe the changes in dietary BCAAs intake in Chinese adults from 1997 to 2015 and the risk of T2D caused by BCAAs intake.

## Methods

### Study Population

All datasets used in this study were from two independent national project, CHNS and CNHS. CHNS was an international collaborative project cohosted by the Carolina Population Center at the University of North Carolina at Chapel Hill and the National Institute for Nutrition and Health (NINH) at Chinese Center for Disease Control and Prevention (CCDC), which aimed to examine the effects of the healthand nutrition. CNHS was a national survey conducted by the CCDC to survey the national health and nutrition status. The sampling method, dietary survey method, anthropometric measurement method, and quality control method of CNHS are almost identical to those of CHNS in terms of cross-section. The provincial staff for both projects are the same team. The core structure of the two surveys is the same in terms of cross-section. Both projects used stratified, multistage, random cluster sampling method, and further detailed information could be referred elsewhere (12, 13).

In the dietary BCAA transition trend analysis, seven cross-sectional data were obtained from CHNS (1997), CHNS (2000), CHNS (2004), CHNS (2006), CHNS (2009), CHNS (2011) and CNHS (2015). Data were included for analysis if dietary intake records were available and the age of the study object was 18 years or older at the time of survey. And data of 9,404, 10,291, 9,682, 9,553, 9,811, 12,686 and 71,695 participants in 1997, 2000, 2004, 2006, 2009, 2011, and 2015 were used for analysis, respectively.

In the BCAAs risk analysis, prospective cohort data were extracted from CHNS (1997–2015). Participants diagnosed with diabetes at baseline, those aged <18 years, and those without dietary records were excluded for analysis, and 15,508 participants with 9.9 ± 5.6 (mean ± SD) follow-up years were finally included for analysis.

### Dietary BCAA Intake Assessment

BCAAs intake were calculated from 24-h dietary recall records and household condiment weighing records for three consecutive days (2 working days and 1 weekend). All field staff are professionally trained nutritionists who work in nutrition in their own county. BCAAs intakes was estimated by multiplying the consumed grams of each food by the amino acid contents of each food (referred from Chinese Food Composition Tables) (14–16) before BCAAs intake for all food items was summed by individual.

In the BCAA risk analysis, dietary exposure to BCAAs were calculated by using the average BCAAs intake values in each record before the onset of diabetes.

### Identification of the New-Onset Diabetes

Since 1997, participants have been asked to report their previous diabetes history in the form of questionnaire interviews at each follow-up. Three questions were used to identify the new onset diabetes in the CHNS project: (1) Have the doctor told you that you suffer from type 2 diabetes? (2) How old (age) were you when this happened? (3) Have you used the following treatment methods, such as special diet, weight control, oral medication, insulin injection, Chinese medicine, etc.? The diagnosis of T2D was based on patient-reported physicians' diagnoses and/or the presence of diabetes-specific medication.

### Statistical Analysis

**Figure 1:**
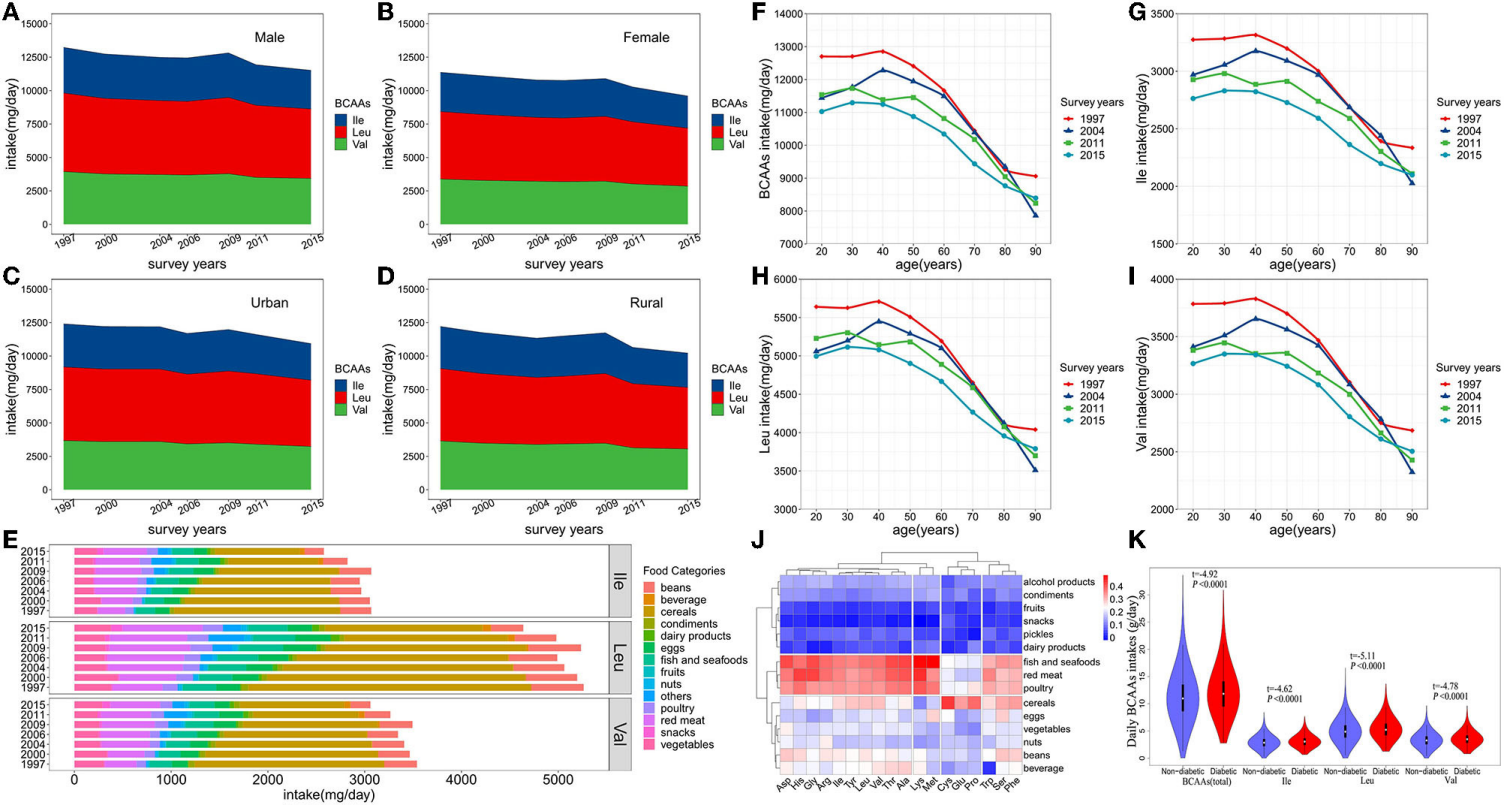
**(A–D)** Trends in consumption of BCAAs and food sources from 1997 to 2015. *P* value for the trend, *P* < 0.0001 (Male, Female, Urban, Rural). **(E)** Food sources of dietary BCAAs. **(F–I)** Average daily BCAAs (Ile, Leu and Val) intakes in adults (aged 18–90 years) during 1997-2015. **(J)** Clustering and correlation heat map between dietary amino acids and food categories. **(K)** Comparison of BCAAs intakes between the diabetic onset group and non-onset group in the cohort.

We provided the demographic characteristics of each survey year. We also calculated the mean (SD) of dietary BCAAs by sex, age group and urban/rural status. A generalized linear model was used to test trends for consumption of BCAAs from 1997 to 2015, adjusting for sex, age, BMI and region. Heatmaps were generated and clustered using hierarchical clustering. For the comparison between the two groups, *t*-test was applied in Figure 1K, and generalized linear model was used in **Table 4**.

Based on the Cox proportional hazard model, a restricted cubic spline (RCS) curve was used to assess the association between dietary BCAAs levels and T2D risk on a continuous scale. In the statistical analyses, we adjusted for age, sex, energy intake, BMI, region, smoking status (previous or present, never), alcohol consumption (yes, no), which were well known risk factors for diabetes. In the Cox proportional hazards models, participants with previously diagnosed diabetes, were excluded when first entry into the survey. To balance best fit and overfitting in the main splines for incident diabetes, the number of knots, between three and six, was chosen as the lowest value for the Akaike information criterion, but if within two of each other for different knots, the lowest number of knots was chosen (17). In the non-linearity test, *P* < 0.1 was considered statistically significant for data exploration and visualization. Otherwise, two-sided significance tests were used throughout, and a two-sided *P* < 0.05 was considered statistically significant. All analyses were conducted using SAS software, version 9.4 (SAS Institute, Cary, NC) and R software, version 4.1.2.

### Patient and Public Involvement

Participants were not involved in setting the research question or the outcome measures, nor were they involved in the design or implementation of the study. No participants were asked to advise on interpretation or writing of the manuscript.

## Results

From 1997 to 2015, the number of participants in the survey increased from 9,404 to 71,695, and the proportion of the elderly and urban residents continued to increase, reflecting increasing trends of aging and urbanization in China (Table 1). From 1997 to 2015, there was a significant decreasing trend in the BCAAs intake of Chinese adults in all subgroups (including the type of BCAAs, age subgroups, sex and urbanization status) (*P* < 0.0001) except for Leu in 80 or older, and a decreasing trend in the consumption of BCAAs after 40 years old (*P* < 0.05) (Table 2; Figure 1). From 1997 to 2015, cereals continued to be the first primary source for dietary BCAA intake, but the proportion of its contribute decreased from 55.6% to 34.9%. Similarly, beans decreased from 10.1 to 7.2%. In contrast, the percent contribution of red meat increased from 9.5 to 17.5%. In addition, the contribution of fish and seafoods increased from 6.5 to 8.6%, and eggs increased from 4.7 to 5.4%.

**Table 1:** Sociodemographic distribution of participants in the 1997962015.

	**1997**	**2000**	**2004**	**2006**	**2009**	**2011**	**2015**
Total	9,404	10,291	9,682	9,553	9,811	12,686	71,695
**Age group (years)**							
18–34	3,339 (35.5)	3,019 (29.3)	2,142 (22.1)	1,780 (18.6)	1,692 (17.3)	2,090 (16.5)	8,695 (12.1)
35–49	3,010 (32.0)	3,636 (35.3)	3,237 (33.4)	3,156 (33.0)	3,122 (31.8)	3,967 (31.3)	18,782 (26.2)
50–64	1,949 (20.7)	2,319 (22.5)	2,782 (28.7)	2,989 (31.3)	3,208 (32.7)	4,278 (33.7)	27,697 (38.6)
65–79	956 (10.2)	1,157 (11.2)	1,333 (13.8)	1,412 (14.8)	1,518 (15.5)	2,004 (15.8)	14,739 (20.6)
80 or older	150 (1.6)	160 (1.6)	188 (1.9)	216 (2.3)	271 (2.8)	347 (2.7)	1,782 (2.5)
**Sex (%)**							
Male	4,562 (48.5)	4,980 (48.4)	4,614 (47.7)	4,538 (47.5)	4,676 (47.7)	5,933 (46.8)	34,140 (47.6)
Female	4,842 (51.5)	5,311 (51.6)	5,068 (52.3)	5,015 (52.5)	5,135 (52.3	6,753 (53.2)	37,555 (52.4)
**Living area (%)**							
Urban	2,971 (31.6)	3,256 (31.6)	3,007 (31.1)	2,984 (31.2)	3,082 (31.4)	5,281 (41.6)	29,145 (40.7)
Rural	6,433 (68.4)	7,035 (68.4)	6,675 (68.9)	6,569 (68.8)	6,729 (68.6)	7,405 (58.4)	42,550 (59.4)

**Table 2:** Trends in mean BCAAs intake among Chinese adults from 1997962015.

		**1997**	**2000**	**2004**	**2006**	**2009**	**2011**	**2015**	*P* for trend*	**Δ**
	**BCAAs (mg/day)**	**Mean (SD)**	**Mean (SD)**	**Mean (SD)**	**Mean (SD)**	**Mean (SD)**	**Mean (SD)**	**Mean (SD)**		
Total	Ile	3165.81 (1124.99)	3102.89 (1122.43)	3004.50 (1189.17)	3018.33 (1172.68)	3063.48 (1181.62)	2805.68 (1159.1)	2635.44 (3301.12)	<0.0001	530.37
	Leu	5449.48 (1995.36)	5277.97 (1947.35)	5140.51 (2076.7)	5115.99 (2036.53)	5261.45 (2065.60)	4995.45 (2110.14)	4746.61 (4820.84)	<0.0001	702.87
	Val	3657.37 (1282.00)	3520.52 (1242.34)	3456.76 (1344.52)	3427.45 (1305.43)	3489.38 (1313.68)	3248.66 (1314.82)	3128.64 (4268.14)	<0.0001	528.73
	BCAAs	12272.66 (4363.69)	11901.38 (4280.58)	11601.77 (4573.22)	11561.77 (4487.12)	11814.32 (4532.28)	11049.79 (4554.87)	10510.69 (12334.01)	<0.0001	1761.97
**Age group (years)**										
18-34	Ile	3277.85 (1127.07)	3174.12 (1128.90)	3015.09 (1157.40)	3054.45 (1149.93)	3206.70 (1228.80)	2965.23 (1196.04)	2812.25 (1441.48)	<0.0001	465.60
	Leu	5627.67 (2006.46)	5375.61 (1939.71)	5129.59 (2006.18)	5190.55 (1985.61)	5519.91 (2148.54)	5284.22 (2157.42)	5084.05 (2633.88)	<0.0001	543.62
	Val	3786.87 (1288.29)	3592.22 (1245.41)	3465.16 (1304.44)	3473.67 (1277.97)	3646.99 (1361.33)	3426.45 (1360.96)	3327.38 (1702.73)	<0.0001	459.49
	BCAAs	12692.39 (4379.5)	12141.95 (4283.15)	11609.85 (4430.17)	11718.68 (4383.86)	12373.61 (4712.2)	11675.9 (4684.62)	11223.68 (5755.53)	<0.0001	1468.71
35-49	Ile	3290.14 (1125.94)	3226.16 (1099.01)	3159.97 (1200.06)	3171.33 (1176.59)	3192.13 (1132.73)	2891.4 (1154.24)	2791.73 (1454.93)	<0.0001	498.41
	Leu	5659.13 (1990.15)	5489.12 (1906.01)	5415.63 (2096.72)	5366.09 (2035.04)	5470.76 (1985.76)	5149.94 (2105.13)	5026.23 (2643.82)	<0.0001	632.90
	Val	3800.32 (1280.56)	3663.71 (1214.83)	3637.73 (1353.41)	3593.17 (1310.39)	3630.29 (1258.27)	3348.39 (1306.01)	3306.51 (1714.99)	<0.0001	493.81
	BCAAs	12749.59 (4359.19)	12378.99 (4186.5)	12213.33 (4611.5)	12130.59 (4495.47)	12293.18 (4345.43)	11389.73 (4535.5)	11124.47 (5789.78)	<0.0001	1625.12
50-64	Ile	3081.67 (1089.61)	3070.33 (1119.17)	3026.88 (1197.89)	3066.46 (1156.92)	3102.08 (1188.87)	2811.08 (1140.81)	2651.93 (5023.03)	<0.0001	429.74
	Leu	5325.64 (1947.39)	5243.37 (1973.22)	5191.05 (2102.94)	5195.02 (2022.77)	5343.00 (2074.06)	5015.5 (2080.58)	4766.21 (7085.95)	<0.0001	559.43
	Val	3565.45 (1242.93)	3489.68 (1242.32)	3488.43 (1358.41)	3486.48 (1284.42)	3542.52 (1322.31)	3258.84 (1294.74)	3156.31 (6561.03)	<0.0001	409.14
	BCAAs	11972.77 (4237.81)	11803.38 (4299.01)	11706.36 (4620.93)	11747.96 (4433.15)	11987.6 (4554.36)	11085.43 (4485.39)	10574.45 (18615.04)	<0.0001	1398.32
65-79	Ile	2693.07 (1012.55)	2709.12 (1066.37)	2666.63 (1105.89)	2653.19 (1127.01)	2699.41 (1105.36)	2561.84 (1123.27)	2357.05 (1164.32)	<0.0001	336.02
	Leu	4656.38 (1810.65)	4617.87 (1857.61)	4558.38 (1935.65)	4506.80 (1974.96)	4621.23 (1936.37)	4536.59 (2048.59)	4254.54 (2153.32)	<0.0001	401.84
	Val	3103.42 (1139.38)	3074.82 (1183.03)	3056.87 (1248.90)	3014.44 (1260.65)	3073.02 (1229.80)	2965.56 (1271.8)	2798.81 (1353.3)	<0.0001	304.61
	BCAAs	10452.88 (3933.61)	10401.81 (4083.21)	10281.88 (4256.11)	10174.43 (4338.58)	10393.67 (4245.9)	10064 (4416.96)	9410.4 (4642)	<0.0001	1042.48
80 or older	Ile	2282.67 (900.67)	2276.84 (964.06)	2271.67 (1057.54)	2206.28 (988.86)	2269.76 (1035.85)	2206.43 (1006.53)	2171.86 (1166.13)	0.0001	110.81
	Leu	3939.90 (1536.34)	3912.45 (1682.44)	3907.61 (1833.79)	3735.89 (1733.01)	3857.34 (1755.69)	3892.75 (1822.17)	3918.36 (2169.47)	0.0979	21.54
	Val	2631.25 (987.66)	2583.53 (1058.60)	2611.61 (1189.12)	2508.16 (1097.33)	2585.18 (1146.85)	2546.96 (1138.18)	2582.16 (1358.49)	0.0304	49.09
	BCAAs	8853.82 (3397.75)	8772.81 (3688.53)	8790.9 (4055.09)	8450.34 (3805)	8712.28 (3920.74)	8646.13 (3944.96)	8672.37 (4668.85)	0.0174	181.45
**Sex**										
Male	Ile	3409.84 (1168.50)	3321.68 (1145.03)	3233.38 (1210.77)	3249.92 (1213.03)	3327.02 (1210.04)	3033.45 (1189.13)	2891.54 (4583.11)	<0.0001	518.30
	Leu	5880.03 (2082.90)	5648.08 (1986.64)	5534.46 (2133.91)	5505.95 (2113.17)	5706.89 (2121.96)	5393.28 (2159.4)	5195.51 (6539.97)	<0.0001	684.52
	Val	3943.19 (1332.62)	3768.90 (1267.69)	3721.41 (1374.79)	3690.08 (1350.08)	3785.64 (1342.90)	3509.78 (1345.81)	3431.11 (5964.2)	<0.0001	512.08
	BCAAs	13233.07 (4541.18)	12738.66 (4366.18)	12489.25 (4677.37)	12445.96 (4646.04)	12819.56 (4642.75)	11936.51 (4662.03)	11518.16 (17038.23)	<0.0001	1714.91
Female	Ile	2935.88 (1030.95)	2897.74 (1060.69)	2796.13 (1129.64)	2808.77 (1093.56)	2823.50 (1101.69)	2605.57 (1093.67)	2402.64 (1263.12)	<0.0001	533.24
	Leu	5043.83 (1818.40)	4930.93 (1843.63)	4781.85 (1955.57)	4763.11 (1896.92)	4855.83 (1925.45)	4645.92 (2001.83)	4338.53 (2266.43)	<0.0001	705.30
	Val	3388.08 (1170.32)	3287.61 (1171.29)	3215.81 (1269.37)	3189.80 (1215.85)	3219.60 (1225.85)	3019.24 (1242.58)	2853.68 (1510.68)	<0.0001	534.40
	BCAAs	11367.79 (3983.52)	11116.29 (4044.61)	10793.8 (4321.02)	10761.68 (4180.55)	10898.93 (4226.6)	10270.74 (4310.95)	9594.84 (4976.1)	<0.0001	1772.95
**Region**										
Urban	Ile	3218.94 (1116.88)	3184.62 (1104.34)	3158.1 (1227.17)	3046.42 (1137.22)	3103.34 (1164.94)	2938.09 (1193.25)	2738.25 (1387.03)	<0.0001	480.69
	Leu	5510.39 (1956.58)	5428.92 (1904.09)	5424.62 (2118.00)	5217.25 (1992.88)	5366.65 (2051.88)	5271.77 (2163.02)	4957.73 (2399.85)	<0.0001	552.66
	Val	3676.81 (1260.35)	3595.33 (1218.96)	3605.08 (1382.04)	3427.72 (1260.11)	3511.99 (1285.59)	3406.9 (1361.22)	3235.19 (1698.24)	<0.0001	441.62
	BCAAs	12406.13 (4306.65)	12208.87 (4205.73)	12187.8 (4699.57)	11691.39 (4365.89)	11981.98 (4477.58)	11616.77 (4693.54)	10931.17 (5348.8)	<0.0001	1474.96
Rural	Ile	3141.27 (1127.96)	3065.06 (1128.79)	2935.31 (1165.14)	3005.58 (1188.31)	3045.23 (1188.82)	2711.25 (1124.71)	2565.02 (4126.97)	<0.0001	576.25
	Leu	5421.35 (2012.54)	5208.11 (1963.25)	5012.52 (2045.13)	5069.99 (2054.55)	5213.27 (2070.22)	4798.38 (2049.12)	4602 (5929.87)	<0.0001	819.35
	Val	3648.4 (1291.88)	3485.90 (1251.59)	3389.94 (1321.95)	3427.32 (1325.60)	3479.02 (1326.32)	3135.8 (1268.79)	3055.66 (5357.86)	<0.0001	592.74
	BCAAs	12211.02 (4388.75)	11759.07 (4307.65)	11337.77 (4490.58)	11502.89 (4540.22)	11737.53 (4555.39)	10645.43 (4409.33)	10222.68 (15379.57)	<0.0001	1988.34

*SD, standard deviation. Linear trends in the mean BCAAs intake from 1997 to 2015 were tested using generalized linear model adjusted for sex, age, BMI and region. Δ1997–2015*.

As shown in Figure 1J, the types of food were clustered into three major groups. Fish and seafoods, red meat and poultry were clustered into one category. Cereals, eggs, vegetables, nuts, beans, and beverages were clustered into one category. Additionally, alcohol products, condiments, fruits, snacks, pickles and dairy products were clustered into one category. Dietary BCAAs (Leu, Ile, and Val) were clustered together with aspartate (Asp), histidine (His), glycine (Gly), arginine (Arg), threonine (Thr) and alanine (Ala). Furthermore, the top 4 types of food, exhibiting the strongest correlation with dietary BCAAs, were fish and seafoods, red meat, poultry and cereals.

At the endpoint of observation, mean BCAAs intake was higher in participants with new-onset diabetes onset than in non-diabetic participants (*t* = −4.92, *P* < 0.0001) (Figure 1K). The same phenomenon were also observed in Ile (*t* = −4.62, *P* < 0.0001), Leu (*t* = −5.11, *P* < 0.0001) and Val (*t* = −4.78, *P* < 0.0001).

**Figure 2:**
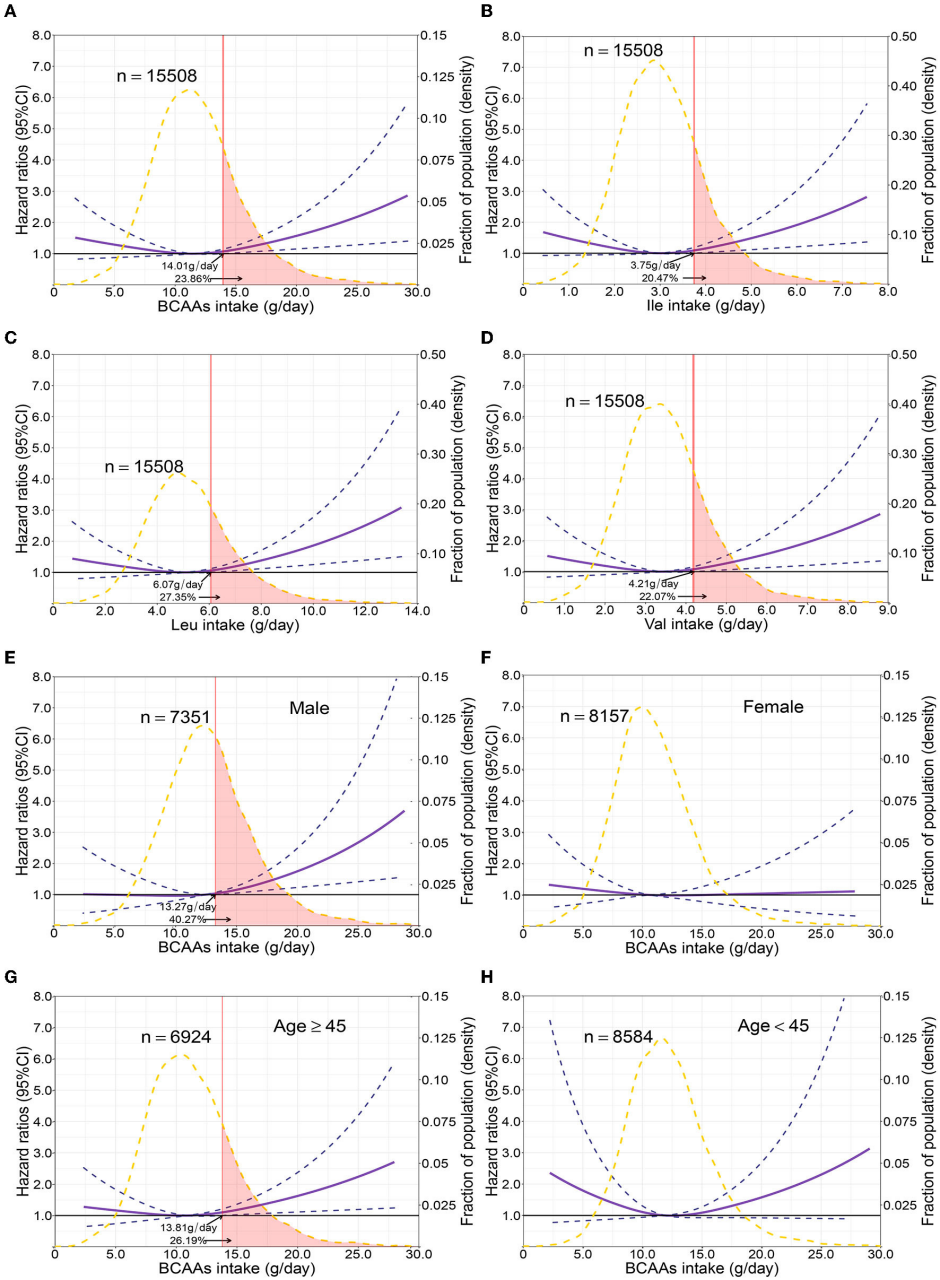
Multivariable adjusted hazard ratios of incident type 2 diabetes according to levels of BCAAs consumption on a continuous scale in the overall population. Solid blue lines are multivariable adjusted hazard ratios, with dashed blues lines showing 95% confidence intervals derived from restricted cubic spline regressions with three knots. Reference lines for no association are indicated by solid bold lines at a hazard ratio of 1.0. Dashed yellow curves show fraction of population with different levels of BCAAs intake. Arrows indicate the lowest consumption of BCAAs and fraction of population with risk of T2D. Analyses were adjusted for age, sex, smoking status, alcohol consumption, BMI, physical activity levels and energy intake at baseline. Based on individuals from the CHNS followed for a mean 9.9 years. **(A–D)** Representation of restricted cubic spline cox regression models for dietary BCAAs, Ile, Leu, Val and risk of type 2 diabetes. **(E–H)** Representation of restricted cubic spline cox regression models for dietary BCAAs and risk of type 2 diabetes in different age and gender subgroups.

The mean intake of BCAAs in the population of cohort study was 11.83 ± 3.77 g/day (Table 3). The impact of dietary BCAA intake on risk of T2D was shown in Figure 2. The consumption of BCAAs and risk of T2D was U-shape-associated and higher dietary BCAAs (**≥** 14.01 g/day) increased the risk of T2D. When upon a closer look, higher intake of each BCAA also increased the risk of T2D (Figure 2). The 95% confidence interval (CI) was above the HR of 1.0, when the consumptions were higher than 14.01, 3.75, 6.07, 4.21 g/day in BCAAs, Ile, Leu and Val. Those with higher dietary BCAAs (Group B **≥** 14.01 vs. Group A < 14.01 g/day) also consumed more food in amounts (1616.96 ± 755.83 vs. 1244.92 ± 524.68 g/day, *P* < 0.0001) (Table 4). The average food intake of group A was 1244.92 (95% reference value 216.55 to 2273.29) g/day.

**Table 3:** Baseline characteristics of 15,508 individuals in the CHNS Study.

	**Dietary BCAAs Centile (g/day)**							
	**1st-5th**	**6th-20th**	**21st-40th**	**41st-60th**	**61st-80th**	**81st-95th**	**96th-100th**	**All**
No. of individuals	778 (5.0)	2,330 (15.0)	3,091 (19.9)	3,113 (20.1)	3,096 (20.0)	2,324 (15.0)	776 (5.0)	15,508
Women	547 (70.3)	1,587 (68.1)	1,949 (63.1)	1,624 (52.2)	1,346 (43.5)	836 (36.0)	268 (34.5)	8,157 (52.6)
Age	50.6 (17.3)	48.3 (16.4)	44 (15)	42.6 (14)	41.7 (13.6)	41 (13.2)	41.1 (14)	43.6 (14.9)
Smoker	174 (22.4)	543 (23.3)	803 (26.0)	960 (30.8)	1,115 (36.0)	942 (40.5)	327 (42.1)	4,864 (31.4)
Drinker	177 (22.8)	557 (23.9)	847 (27.4)	1,072 (34.4)	1,299 (42.0)	1,071 (46.1)	397 (51.2)	5,420 (34.9)
Height	157.5 (8.3)	158.3 (8.4)	159.6 (8.2)	161.1 (8.2)	162.4 (8)	163.9 (8.2)	165.7 (7.8)	161.1 (8.5)
Weight	57.3 (11.5)	57.3 (11.1)	58.4 (10.7)	59.3 (10.7)	60.5 (10.3)	62.6 (11.2)	64.7 (11.6)	59.7 (11.1)
BMI	23 (3.9)	22.8 (3.5)	22.9 (3.4)	22.8 (3.2)	22.9 (3.2)	23.3 (3.5)	23.5 (3.5)	22.9 (3.4)
Systolic blood pressure (mm Hg)	125.4 (20.3)	121.9 (19.4)	119.4 (18.1)	118.9 (16.9)	118.4 (15.4)	119.4 (15.7)	120.9 (15.3)	119.9 (17.3)
Diastolic blood pressure (mm Hg)	79.1 (11.6)	77.8 (11)	77.4 (11.1)	77.3 (10.7)	77.3 (10)	77.8 (10.3)	78.6 (10.9)	77.6 (10.7)
Triceps skin fold (mm)	16.5 (8.1)	15.5 (8)	15 (8.1)	14.5 (7.9)	14.5 (8)	15.3 (8.4)	16.6 (8.6)	15.1 (8.1)
Hip Circumference (cm)	92.7 (9)	92.5 (8.5)	92.8 (8.4)	92.7 (8.3)	93.1 (8)	93.9 (8.2)	94.8 (9.5)	93 (8.4)
Waist Circumference (cm)	80.6 (10.6)	79.5 (10.2)	79.3 (10.6)	79.3 (9.9)	79.6 (9.9)	81 (10.4)	82.6 (11)	79.9 (10.3)
Upper Arm Circumference (cm)	26.3 (4.5)	26.0 (4.3)	26.0 (3.9)	26.2 (4)	26.4 (4)	26.9 (4)	27.9 (5.9)	26.4 (4.2)
BCAAs intake(g/day)	5.14 (1.18)	7.81 (0.66)	9.75 (0.52)	11.49 (0.49)	13.37 (0.61)	16.08 (1.08)	21.44 (2.72)	11.83 (3.77)

*Values are means (standard deviation) or number (%). BCAAs, Branched-Chain Amino Acids*.

**Table 4:** Differences in diet with upper and lower thresholds of BCAAs.

**Variables**	**Group A BCAAs <14.01 g/day (*n* = 11,808)**	**Group B BCAAs ≥14.01 g/day (*n* = 3,700)**	**χ^2^/F**	***P-value***
	***n*** **(%)/Mean (SD)**	***n*** **(%)/Mean (SD)**		
**Demographic characteristics**				
Female	6,800 (57.59%)	1,357 (36.68%)	494.1568	<0.0001
Age	44.43 (15.16)	41.07 (13.48)	12.85	<0.0001
≥60 years	2,161 (18.30%)	386 (10.43%)	127.0792	<0.0001
BMI	22.85 (3.37)	23.25 (3.45)	−6.03	<0.0001
Smoking history	3,371 (28.55%)	1,493 (40.35%)	182.3113	<0.0001
Alcohol consumption history	3,685 (31.21%)	1,735 (46.89%)	304.8290	<0.0001
Energy intake	2,042.12 (707.20)	2593.34 (1215.11)	−26.23	<0.0001
**Food categories (g/day)**				
Cereals	451.00 (194.16)	526.54 (262.78)	206.97	<0.0001
Beans	53.51 (64.61)	84.63 (90.13)	329.59	<0.0001
Vegetables	393.83 (196.80)	453.22 (253.42)	166.45	<0.0001
Pickles	3.39 (10.05)	3.77 (10.34)	7.47	0.0063
Fruits	59.74 (104.03)	75.91 (125.65)	27.23	<0.0001
Nuts	4.24 (14.08)	8.69 (23.29)	149.07	<0.0001
Red meat	74.94 (68.94)	121.36 (106.93)	568.14	<0.0001
Poultry	13.75 (29.13)	30.87 (52.40)	445.93	<0.0001
Dairy products	17.19 (58.41)	37.23 (91.20)	169.43	<0.0001
Eggs	31.09 (34.04)	45.86 (49.85)	247.45	<0.0001
Fish and seafoods	29.49 (45.91)	66.49 (81.14)	982.46	<0.0001
Snacks	11.24 (37.46)	15.43 (51.95)	8.52	0.0035
Sugar and starch	2.91 (10.40)	3.87 (13.70)	6.75	0.0094
Sauce	0.52 (3.12)	0.43 (2.65)	2.27	0.1321
Alcohol products	10.93 (55.62)	27.27 (104.20)	58.74	<0.0001
Fast food	10.32 (37.51)	17.08 (62.32)	17.47	<0.0001
Beverage	3.26 (60.59)	9.84 (58.11)	21.2	<0.0001
Vegetable oil and condiments	56.17 (32.71)	64.25 (35.98)	72.53	<0.0001
Others	17.43 (38.21)	24.21 (64.06)	26.63	0.0004
Total food intake	1244.92 (524.68)	1616.96 (755.83)	671.59	<0.0001

*SD, standard deviation. Demographic characteristics were tested using Chi-square test and student's t-test. Group differences of food consumption were calculated using the generalized linear models after adjusting for age, sex, energy intake, BMI, region, physical activity levels, smoking status (previous or present, never), alcohol consumption (yes, no)*.

Compared with individuals with BCAAs consumption of 10.65–12.37 g/day, the multivariable-adjusted HR for diabetes was 2.26 (95% CI 1.45 to 3.51) for individuals with consumption of BCAAs more than 18.52 g/day (Table 5). The same trends were found in Ile and Leu, except for Val. The results were unaffected by multivariable adjustments in BCAAs, Ile and Leu.

**Table 5:** Hazard ratios for incident type 2 diabetes according to categories of levels of BCAAs (Ile, Leu, Val) intake, sex and age adjusted, and multivariable adjusted.

**Centile**	**Consumption (g/day)**	**Individuals**	**Events**	**Event rate per 1,000 person years**	**Age and sex adjusted hazard ratio (95% CI)**	**Hazard ratio (95% CI)**	**Multivariable adjusted hazard ratio (95% CI)**	**Hazard ratio (95% CI)**
**BCAAs**								
1st-5th	<6.44	778	21	4.98	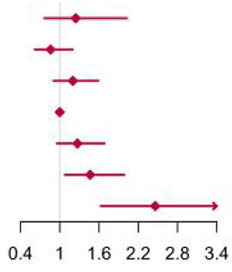	1.25 (0.77–2.03)	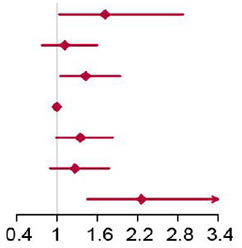	1.72 (1.03–2.88)
6th-20th	6.44–8.82	2,330	61	2.97		0.86 (0.62–1.20)		1.12 (0.78–1.6)
21st-40th	8.82–10.65	3,091	108	3.32		1.20 (0.91–1.59)		1.43 (1.05–1.95)
41st-60th	10.65–12.37	3,113	90	2.54		1.0		1.0
61st-80th	12.37–14.53	3,096	106	3.05		1.27 (0.96–1.68)		1.35 (0.99–1.84)
81st-95th	14.53–18.52	2,324	79	3.38		1.47 (1.08–1.99)		1.27 (0.9–1.78)
96th-100th	>18.52	776	31	5.56		2.46 (1.63–3.72)		2.26 (1.45–3.51)
**Ile**								
1st-5th	<1.64	769	22	5.41	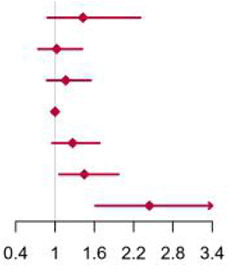	1.43(0.88–2.30)	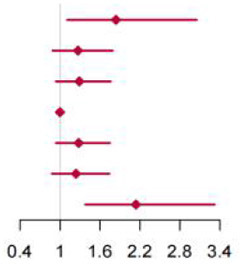	1.84 (1.11–3.05)
6th-20th	1.64–2.27	2,332	68	3.38		1.03 (0.74–1.42)		1.27 (0.89–1.79)
21st-40th	2.27–2.75	3,069	103	3.20		1.16 (0.88–1.55)		1.29 (0.94–1.76)
41st-60th	2.75–3.20	3,124	89	2.50		1.0		1.0
61st-80th	3.20–3.77	3,131	106	3.01		1.27 (0.96–1.68)		1.28 (0.94–1.75)
81st-95th	3.77–4.77	2,303	77	3.26		1.45 (1.06–1.97)		1.24 (0.88–1.74)
96th-100th	>4.77	780	31	5.43		2.44 (1.62–3.69)		2.14 (1.38–3.32)
**Leu**								
1st-5th	<2.83	783	19	4.34	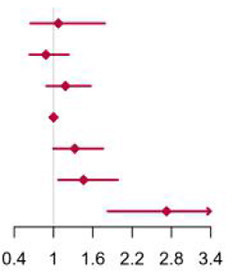	1.07 (0.65–1.78)	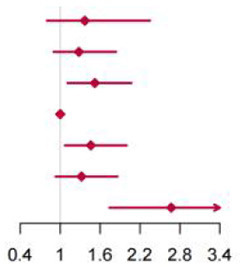	1.37 (0.80–2.35)
6th-20th	2.83–3.88	2,305	62	3.02		0.88 (0.63–1.23)		1.28 (0.90–1.84)
21st-40th	3.88–4.72	3,126	107	3.22		1.18 (0.89–1.57)		1.52 (1.11–2.07)
41st-60th	4.72–5.51	3,110	89	2.51		1.0		1.0
61st-80th	5.51–6.49	3,081	107	3.13		1.32 (1.00–1.76)		1.46 (1.07–2.00)
81st-95th	6.49–8.34	2,330	78	3.37		1.46 (1.07–1.98)		1.32 (0.93–1.86)
96th-100th	>8.34	773	34	6.16		2.72 (1.83–4.06)		2.67 (1.74–4.11)
**Val**								
1st-5th	<1.93	785	21	4.96	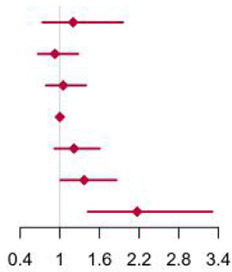	1.20 (0.74–1.95)	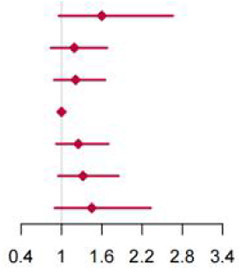	1.60 (0.96–2.66)
6th-20th	1.93–2.62	2,323	67	3.33		0.93 (0.67-1.28)		1.19 (0.84–1.68)
21st-40th	2.62–3.16	3,117	101	3.08		1.05 (0.79–1.39)		1.21 (0.89–1.65)
41st-60th	3.16–3.66	3,062	93	2.67		1.0		1.0
61st-80th	3.66–4.30	3,131	108	3.06		1.21 (0.92-1.60)		1.25 (0.92–1.70)
81st-95th	4.30–5.44	2,314	77	3.28		1.37 (1.01–1.86)		1.32 (0.95–1.85)
96th-100th	>5.44	776	29	5.13		2.17 (1.43–3.31)		1.45 (0.90–2.33)

*Multivariable adjusted analyses were adjusted for age, sex, smoking status, alcohol consumption, BMI, physical activity levels and energy intake at baseline. Based on individuals from the CHNS followed for a mean 9.9 years. Interaction with consumption of red meat (P-value for interaction > 0.05), fish and sea foods (P-value for interaction > 0.05), poultry (P-value for interaction > 0.05)*.

### Sensitivity Analyses

When fractional polynomials was applied, the U-shaped association between dietary BCAAs intake and T2D risk also exist, and the BCAAs consumption cut-off that increased the T2D risk was 18.52 g/day (Table 5). When further stratified by sex and age, the association between the two was unaltered in men or in participants aged 45 years and older. However, the association between BCAAs intake and risk of T2D diminished in females or in participants younger than 45 years (Figures 2E–H).

## Discussion

Using seven large-scale nationally representative survey data, a decreasing trend in dietary BCAAs intake was observed in the study population at all ages from 1997 to 2015. Consumption of BCAAs also declined as age increased for those aged 40 years older. In all food categories, the strongest correlations with BCAAs were with red meat, poultry, fish and seafoods. And the risk analysis showed that increased BCAAs intake was associated with an elevated risk of T2D. This association was more stable among men and among people with middle-age and elder. The people with risk of T2D accounted for about 23.86% of the total population due to BCAAs.

To the best of our knowledge, this is the largest study including the most recent national survey data to first address the dietary BCAA intake trend and its risk on T2D. The reliability of our result could be guaranteed by the strict quality control of the CHNS and CNHS, including standardized protocols, standardized data collection procedures and standardized training of the field working stuff. This study contributes to the discovery of the relationship between dietary BCAAs and chronic diseases in the Chinese population.

The declines in BCAAs intake may well have contributed to the declining T2D morbidity. According to a recent study, the incidence of diabetes decreased from 2007 to 2017 in both men and women in China (18). And, the trend in consumption of BCAAs paralleled with the decreased trend of T2D incidence, which reduced by 14.36% from 1997 to 2015 in the adult population (Table 2). The declined BCAAs intake reflected changes of society and behavioral lifestyle in China. Accompanying with the decreasing BCAAs consumption, it was also observed that energy and protein intake decreased substantially from 1992 to 2012 among Chinese adults (11). One possible reason for these declines could be decreased physical activity. In China, although leisure-time physical activity have generally increased since 2000 (19), total physical activity have dropped sharply from 1991 to 2009 (20), and classical literatures showed a J-shaped relationship between physical activity and energy intake (21, 22). However, physical activity was also inversely related to incident diabetes (23). Still, the age-standardized incidence rates of diabetes subsequently decreased from 2007 to 2017 (18).

In dietary BCAA risk analysis of the cohort, increased dietary BCAAs intake was associated with an elevated risk of T2D. Men and older people were more sensitive to the risk of diabetes caused by BCAAs. The conclusions reached in this study were similar to previous studies in the US and northeastern China (2, 7, 8). In the prospective cohort study of United States, HR of diabetes for the highest quintile of BCAAs intake compared with the lowest quintile were 1.13 (95%CI, 1.07–1.19, *P* < 0.001) in leucine, 1.13 (95%CI, 1.07–1.19, *P* < 0.001) in isoleucine and 1.11 (95%CI, 1.05–1.17, *P* < 0.001) in valine (2). In Harbin, China and the American population, it has been observed that higher dietary BCAA intake will promote the risk of T2D. The Harbin population study showed that the OR and 95% CI across quartiles of total BCAA intakes for T2D within the 4th quartile were 1.0, 1.337 (0.940–1.903); 1.579 (1.065–2.343); 2.412 (1.474–3.947) (8). In a meta-analysis study, higher total intake of BCAAs causes increased T2DM risk with an OR and 95% CI of 1.32 (1.14, 1.53) (24). However, the results may seem in contrast to the study from Japan (10). The Japanese study showed that increased intake of BCAAs may be associated with a reduced risk of diabetes. The HR between the highest tertile and the lowest tertile was 0.70 (95% CI: 0.48–1.02; *P* for trend = 0.06). In that study, total BCAA, leucine and valine intakes were inversely associated with T2D risk in women, and no associations were found in men. Studies have shown that dietary BCAAs affect human metabolism and the risk of chronic diseases (25). A study of young people in northern China showed that a higher dietary BCAA ratio was negatively correlated with postprandial blood glucose (26). Reducing the intake of dietary BCAAs can improve glucose tolerance and body composition (27, 28). Although studies have shown that dietary BCAAs were closely related to multiple chronic diseases, this paper bridges a gap in large cohort studies of representative populations of Chinese.

Of serum BCAAs levels, 80% were determined by protein or BCAAs from diet or supplements, and the remaining 20% are related to their catabolites (29, 30). Studies have shown that oral BCAAs supplementation can affect the leucine content in blood circulation. The relationship between serum BCAAs levels and the occurrence and development of chronic diseases were well established. Studies have found that elevated levels of serum BCAAs are closely related to weight gain, insulin resistance, and abnormal glucose metabolism in adults (31, 32). Animal experiments have shown that in non-obesity, insulin resistance, and fructose-fed rat models, elevated serum BCAA levels were associated with insulin resistance (33). Previous studies also showed higher plasma levels of BCAAs were associated with an increased risk of T2D (34, 35). Prospective population studies have proved that serum BCAA levels can predict the future risk of diabetes (36). In patients with overweight and metabolic syndrome, there was also a correlation between plasma BCAA levels and red meat or animal protein (37). Therefore, control of serum BCAAs can start from dietary BCAAs intake. Our results link dietary BCAAs with population health, especially the risk of diabetes. In this study, group B (BCAAs ≥ 14.01 g/day) was significantly higher than group A (BCAAs < 14.01 g/day) in total food intake and most food categories (*P* < 0.0001), (Table 4). From this point of view, high consumption of BCAAs is accompanied by high consumption of food. Our results are in accordance with a recent study. When the quantity of food intake exceeded certain thresholds, the risks of new-onset diabetes increased or reached a plateau (38).

In all food categories, the strongest correlations with BCAAs were with red meat, poultry, fish and seafoods. Our research found that although BCAA intake is decreasing, sources have changed over time. Now animal sources are main sources and previously cereals. Meanwhile, there was also a correlation between plasma BCAA levels and red meat or animal protein (37). A similar phenomenon was also found in the Brazilian population that the main food sources of BCAA were unprocessed red meat, unprocessed poultry, bread and toast, beans and rice (39). Epidemiological studies have shown that high consumption of animal protein, especially red meat with high levels of methionine and BCAAs, have promoted the progression of age-related diseases (40). And, reducing BCAAs consumption in the Western diet improved glucose tolerance and relieved insulin resistance. Previous research has indicated that reducing dietary BCAAs may represent a highly translatable option for the treatment of obesity and insulin resistance in animals (41). According to the results of this study, we propose dietary recommendations for the population's diet to prevent diabetes. The dietary intake should not exceed 2,273 g/day, and the intake of red meat, poultry, fish and seafoods should be controlled at the same time.

Our study also has several limitations. First, these surveys are not carried out annually, which could have allowed more details in trends. Second, dietary consumption data from the CHNS survey 2015 was not available. We used the dietary information from CNHS survey 2015 for make-up. Statistical processing was used to ensure the quality of the results and the comparability between the CNHS and CHNS. Third, our dietary intake estimates are mainly based on 3-day 24-h meal recall, so measurement errors are inevitable. In order to reduce selection biases and measurement errors, we averaged three 24-h dietary recalls for different age groups or urban/rural areas. The average long-term intakes were used to represent the dietary exposure level of the participants. Finally, when the CHNS survey was planned and implemented, the State Statistical Office of China would not share their sample frame with the CHNS team. Furthermore, the data sets for public distribution would not be released if the CHNS team had worked with them. However, the design used extant census data as best as we could for a multi-level random sample.

In conclusion, a trend toward decreased BCAAs intake was observed in Chinese of all subgroups (including age and sex) from 1997 to 2015. After 40 years of age, consumption of BCAAs declined with increasing age. In all food categories, the strongest correlations with BCAAs were with red meat, poultry, fish and seafoods. Higher BCAAs intake was associated with higher risk of T2D. This relationship is more stable among men and middle-aged and elderly people. The people with risk of T2D accounted for about 23.86% of the total population due to BCAAs. Based on the results of this study, in order to prevent diabetes, we recommend that dietary intake should be restricted, while controlling the intake of red meat, poultry, fish and seafood.

## Data Availability Statement

The datasets presented in this article are not readily available because the copyright of the dataset is currently owned by the Chinese Center for Disease Control and Prevention and has not been fully disclosed yet. Requests to access the datasets should be directed to https://www.cpc.unc.edu/projects/china.

## Ethics Statement

The studies involving human participants were reviewed and approved by National Institute for Nutrition and Health, Chinese Center for Disease Control and Prevention. The patients/participants provided their written informed consent to participate in this study.

## Author Contributions

JZ is guarantor, designed the study, principal investigator, and attests that all the listed authors meet the authorship criteria and that no others meeting the criteria have been omitted. LY conducted the data analysis and drafted the manuscript. PS, QZ, YL, SJ, SZ, and ZW critically revised the manuscript for important intellectual content. All authors contributed to the article and approved the final version of the manuscript.

## Funding

This study was supported by the National Health Commission of the People's Republic of China Medical Reform Major Program: China National Chronic Diseases and Nutrition Surveillance of Adults (2015–2017) and sponsored by National Institute for Nutrition and Health, China CDC project-Research on Dietary and Nutritional Status of Chinese Elderly (No. 150052). This study was also financed by Investigation on frailty and risk factors of the elderly in the community and discussion on the path of nutrition improvement and by Shandong Medical and Health Science and Technology Development Project (SMHSTDP) 2019WS436.

## Conflict of Interest

The authors declare that the research was conducted in the absence of any commercial or financial relationships that could be construed as a potential conflict of interest.

## Publisher's Note

All claims expressed in this article are solely those of the authors and do not necessarily represent those of their affiliated organizations, or those of the publisher, the editors and the reviewers. Any product that may be evaluated in this article, or claim that may be made by its manufacturer, is not guaranteed or endorsed by the publisher.
